# Migraine in childhood: biobehavioural or psychosomatic disorder?

**DOI:** 10.1186/s10194-016-0675-0

**Published:** 2016-09-08

**Authors:** Vincenzo Guidetti, Noemi Faedda, Michael Siniatchkin

**Affiliations:** 1 Department of Pediatric and Child and Adolescent Neuropsychiatry, Sapienza University of Rome, Via dei Sabelli, 108, 00185 Rome, Italy; 2 Department of Medical Psychology and Medical Sociology, Christian-Albrechts-University, Kiel, Germany

**Keywords:** Migraine, Psychological comorbidities, Psychosomatic disorder, Biobehavioural disorder

## Abstract

It is well documented that headache is a multifactorial disorder which includes not only genetic, biological, medical and neuropsychological factor but also psychological and personality traits. The close relationship between stress and migraine attacks and the significant psychiatric comorbidities in migraine provide evidence of a “paradigm” of tight interaction between somatic and psychological aspects in paediatric migraine. In particular in younger children, an uncomfortable situation, a psychological problem or an emotional distress is rarely expressed directly but usually through physical symptoms. So migraine may be considered as a disorder of psychobiological adaptation in which genetic predisposition interplays with internal and/or external environmental influences such as psycho-emotional, climatic, hormonal, dietary or other factors.

In the preface to the 1970 edition of “Migraine” [1], Oliver Sacks says “Some patients I could help with drugs, and some with the magic of attention and interest…it now became apparent to me that many migraine attacks were drenched in emotional significance.” Oliver Sacks recognizes the importance of psychological factors in the pathogenesis of migraine and points to the psychosomatic nature of this disorder. When a child with migraine is referred to a doctor, the doctor may be often surprised by the large amount of accompanying symptoms and comorbidities at the time point of investigation or in the past clinical history: soft neurological signs [2], sleep disturbances [3], allergy [4], problems with school achievement [5], lack of concentration [5], school phobia [5], hyperactivity [6], periodic syndromes [7], anxiety and depression [8, 9], as well as panic attacks [10]. Both the close relationship between stress and migraine attacks and the significant psychiatric comorbidities in migraine provide evidence of a “paradigm” of tight interaction between somatic and psychological aspects in paediatric migraine. This interaction characterizes migraine in children and adolescents as a disorder on the edge between paediatrics and psychiatry. Thus, migraine is one of the typical disorders illustrating an exciting dichotomy between mind and body as well as between health and disease [11].

Migraine may be considered as a disorder of psychobiological adaptation in which genetic predisposition interplays with internal and/or external environmental influences such as psycho-emotional, climatic, hormonal, dietary or other factors [11]. In this context we should postulate that there is a mutual pathogenetic link between migrainous headaches and the loss of the ability to balance homoeostatic changes which occur under the influence of different stressors.

Indeed the role of a maladaptive stress response in migraine has become of great interest in recent years [12, 13]. Studies have focused in particular on the default mode network (DMN), which plays an important role in adaptive behavior and in several cognitive processes, such as memory and social evaluation, the ability to understand the emotions of other people and the ability to reflect about one’s own emotional state. DMN is considered a network associated to individual stressful experiences and coping strategies to promote adaptation (i.e. allostasis) [14–16]. Tessitore et al. [14] demonstrated a decreased functional connectivity within the prefrontal and temporal cortices of the DMN in patients with Migraine without Aura (MwA) during the interictal period. The authors believe that these reduced connectivity in DMN may represent an early migraine biomarker, probably associated to a maladaptive brain response. Moreover Default mode network (DMN), which is impaired in headache patients, plays an important role also in the ability to understand the emotions of other people and the ability to reflect about one’s own emotional state. Indeed recent research found higher levels of alexithymia in headache patients than in controls and alexythimia seems to be associated to a higher risk of developing psychosomatic complaints and psychological problems [17]. Specific changes in connectivity of DMN could be associated to the difficulty to regulate emotions and to express feelings.

Therefore individuals with alexithymia are more likely to have headache and other somatic complaints because they would lack the necessary skills to modulate the emotions and the adequate coping reaction to a stressful situation.

However the link between stress and migraine remains difficult to understand fully.

It is well known that answer to stress is related more to quantity and intensity of individual emotional activation induced by the stimulus or the situation, than to the quality of stressors.

Stress can provoke biological modifications lowering the threshold of the individual’s susceptibility to a migraine attack. As a consequence stress could also precipitate illness in susceptible individuals [18]. Indeed, it seems likely that the well-known hypersensitivity to pain common in the majority of migraine patients, is related more to how these stimuli are individually processed than to their intrinsic nature [19]. In this sense, pain in migraineurs could be considered as the external manifestation of the “alarm” reaction inducing response to the stress.

As an example, a significant increase in the prevalence of headache was found in children from 3 to 11 years of age [20–22] mainly during the transition from preschool years to the elementary school [23]. This change may in fact represent a high stress load and increased requirements on adaptive abilities in a child’s life which may results in homoeostatic unbalances and in headache.

In its original function, pain initiates adaptive processes, like preparation to fight or escape/avoidance reactions, in association with anger or anxiety, by the chronic exposure, may lead to depression [8, 9, 24, 25]. Welch [26] suggested that migraine attacks may results from the functional activation of the sympathetic nervous as an expression of the fight or flight reactions. According to Cannons’ fight or flight theory, the peripheral sympathetic activation during any stressful situation can be attributed to the central perception and processing of stressful events. In migraine patients, this central sensory processing will be initiated in the visual system [26]. Both the sensory input and the autonomous reaction to a significant stimulus are regulated by the frontal cortex. Therefore, migraine attacks seem to occur as a consequence of the imbalance between the brain stem projections upon the intrinsic noradrenergic system and frontal regulatory executive mechanisms resulting in sensory and sympathetic hyper-activation [26]. This theory helps to understand what happens during a migraine attack, but it does not explain why children with migraine are sensible to stressors in the headache-free interval.

Arena and colleagues [27] postulate that the psycho-behavioural characteristics of migraine sufferers are related to an individual emotional-reactive pattern established before the first migraine attack and occur independently of attacks. Guidetti and Ottaviano [28] followed, for over 9 years, 102 full-term newborn healthy infants, who were hyper-reactive in infancy and 80 matched infants, who were not hyper-reactive. Of the 102 children of the first group, 78 (76 %) developed headache or periodic syndromes. This was the case in only 15 (19 %) of the 80 control children. The average age of onset of headache was 7 years. The same authors compared a group of 40 children with MwA with 70 children without headaches for nine temperamental variables (Middle Childhood Temperamental Questionnaire): activity, predictability, approach, adaptability, intensity, mood, persistence, distractibility and threshold. They found that migrainous children had greater hyperactivity and distractibility but lower adaptability, persistence and threshold [2].

Bearing in mind Welch’s hypothesis concerning the significance of frontal function for development of migraine attacks [26], it is worthy to mention that disorders of prefrontal function often result in high distractibility, low persistence, low threshold to stimulation and low adaptability [29]. These observations provide an evidence for the described altered balance between abnormal frontal function, cortical hypersensitivity and sympathetic hyperactivity in children with migraine. Additional evidence of abnormal frontal function in migraineurs may be found in experimental studies which report cognitive impairment in migraine patients in tests of visual attention [30], simple reaction time [31], verbal ability [32] and information processing speed [33].

Magnetic resonance imaging (MRI) found a high prevalence of white matter lesions (WMLs) in patients suffering from migraine which could explain executive deficits. Camarda et al. [34] showed that patients with MwA have cognitive disturbances in tasks assessing executive functions, and that these deficits are associated with the duration and intensity of migraine attacks. Thus they suggested that cognitive dysfunction in migraine may be a consequence of the cumulative effects of repeated migraine attacks leading to subcortical WMLs. However findings are mixed: some studies have not showed differences in executive functions between migraineurs and controls [35]; other studies suggest that the presence of executive deficits in migraine exists, but it is not related to the presence of WMLs [36].

How can the increased sensitivity to stress as well as the psychobehavioral features of migraineurs be explained from a neurophysiological point of view? The most appropriate explanation may be the hypothesis describing abnormal interaction between the Arousal Unit and frontal executive system in migraineurs [37]. The Arousal Unit, which comprises an important group of cells throughout the romboencephalon, mesencephalon and diencephalon, is functionally specialized in the maintenance of the levels of cortical tone (arousal), of attention as well as of perceptual and behavioural reactivity. Therefore, it may influence many behavioural characteristics like adaptability, persistence, threshold to stimulation and, in such a way, temperament. This hypothesis seems to be supported by Rothbart’s [38] definition of temperament that represents “individual differences in reactivity and self-regulation assuming a constitutional basis with a degree of immutability”. It seems likely that in infants the hyperreactivity of the Arousal Unit in combination with the underdeveloped executive control result in the described behavioural and sensory hyperreactivity. In older children and adolescents, the hyperreactivity of the Arousal Unit is balanced by a better executive control and results in the abnormal information processing which was repeatedly described in migraine patients. For example investigating evoked and event-related potentials, Gerber and Schoenen et al. [39, 40] described a deficient habituation, or even potentiation, of cortical responses during repetitive stimulation between attacks in patients suffering from migraine. Habituation is a natural mechanism protecting the brain against overstimulation. The described lack of habituation may result in central overload and neuro-metabolic shift underlying the beginning of the migraine attack. Indeed, the most pronounced deficit of habituation may occur few days before the beginning of an attack; this altered processing reaches its maximum, and then normalizes during the attack [40, 41]. The lack of habituation may be explained by a possible “thalamo-cortical dysrhythmia”, an altered rhythmic stream between thalamus and cortex, or by an abnormal glutamatergic neurotransmission [40, 41]. Whatever the mechanisms, it seems likely that the abnormal processing of cortical information may represent a necessary link between stress, psychological factors and development of migraine attacks.

Gerber, Schoenen et al. [39, 40] described these mechanisms as an initial disturbance of the modulation of sensory stimulus and reaction of the brain which may result from innate hypo-function of state-setting, in particular of serotoninergic brainstem afferents. These abnormalities may be innate, but they could also be due to learning processes, or, alternatively, there could be an interaction between the genetic and learning processes in the development of the abnormal processing of cortical information in migraine.

It is well documented that headache is a multifactorial disorder which includes not only genetic, medical and neuropsychological factor but also psychological and personality traits. Twin studies found that only about half of the variability is explained by “genetic” factors [42]. Epigenetic mechanisms, mainly occurring during early infancy and childhood, play an important role in the pathophysiology of headaches. Child environment, an early stress and the mother-child relationship are important factors of epigenetic changes in the pathogenesis of migraine [42]. This link between genetic and epigenetic, factors is reflected in a peculiar structure of personality that may predispose to headache attacks or may render the brain more susceptible to trigger factors. Competition, perfectionism, ambition, rigidity and tendency to suppress emotions are some of the common psychological and behavioural characteristics of young and adult with headache [43]. Wolff [44] describes migraine patient as rigid, compulsive, perfectionist, ambitious and competitive. They are shy, withdrawn, sober, polite, well-mannered, conscientious, responsible, unusually thoughtful, and extremely obedient to parental wishes [45]. Bille [46] tested 73 children with migraine occurring at least once a month and 73 headache-free controls. Migraineurs described themselves as more anxious, fearful, tense and nervous, while parents described them as more anxious, sensitive, vulnerable to frustrations, more tidy and less physically enduring that did the parents of children without migraine. Kowal and Pritchard [47] confirmed Bille’s data finding in children with headache more psychosomatic problems and behavioural disturbances.

Galli, Guidetti and colleagues (2006) found that children and adolescents with headache were more likely to have other somatic symptoms (e.g. abdominal pain) [48]. Several periodic syndromes of childhood, including abdominal migraine were described like common precursors of migraine. Romanello et al. found [49] a specific association between migraine and infantile colic, suggesting a common pathophysiology of migraine and infantile colic. The pathogenesis of abdominal migraine could be inked to an increased arousal in the central nervous system (CNS) in response to triggers, thus releasing neuropeptides and neurotransmitters that lead to dysregulation of the gastrointestinal system [50]. Furthermore calcitonin-gene-related peptide (CGRP), released during migraine attacks [51] is also potentially involved in the pathogenesis of abdominal pain by inducing the neurogenic inflammation of sensory neurons in the gut [49].

Andrasik [52] confirms these data finding that children that are more depressed express a greater number of somatic complaints and experiencing more internalizing behaviour disorders, such as anxiety and mood disorders. This author noted that one limitation of his study was the lack of a control group of children who had pain from causes other than migraine; therefore he couldn’t rule out the possibility that the personality differences could be the result of chronic pain disorder [53]. Indeed Cunningham [45] compared a group of migrainous children with another with chronic musculoskeletal pain. He did not find any difference in children with different disorders: both groups had somatic complaints including vomiting, nausea, perceptual disturbances and internalizing behaviour problems, all migraine-related phenomena. He suggested that perhaps the personality and behavioural features thought to be characteristic of migraine may instead be related to the experience of chronic pain.

On the other hand, Wayne Holder and others [54] underline the influence of parental behaviour in the maintenance of headache in childhood. Indeed previous studies on children with migraine reported a rigorous and strict familiar environment, demanding discipline with high levels of conventionality, low levels of intimacy and emotional participation [55].

Furthermore many studies have reported that headache in children and adolescents is related to several psychosocial factors, such as maternal depression, psychopathology in childhood, social disadvantage and a family with a history of “painful conditions” [8, 9, 56].

Zuckerman and colleagues [57] found that stomach aces and headaches in pre-school children were strictly associated with maternal depression. Our group tried to focus the attention on the mother-migrainous child link [58], comparing a group of 24 children with migraine without aura to a group of in-patient peers, with a similar chronic pathology with acute exacerbations (recurrent upper respiratory airways infections). The mothers of all patients answered a questionnaire on complete family and personal history to assess the presence of migraine, psychiatric, psychosomatic and neurological disease, use of hypnotic, tranquillizer and antidepressant drugs, and traumatic events. After that a semi-structured interview consisting of 57 items, exploring six different areas (pregnancy, delivery and perinatal period, first year of life of the baby, mother-child relationship after the first year of life, school adjustment, child’s rhythm and the family) was administered to the mothers. The mothers of the migrainous children had significant higher scores for migraine and psychiatric comorbidity (anxiety and depression) and for drug use [57]. They reported more psychological distress during delivery and during the perinatal period and more difficulties to adapt to the child’s needs. The mother-child relationship interferes on peculiar temperamental component of the child and vice-versa, and it plays a role in the occurrence of migraine attacks [59].

Several studies describe the influence of maternal psychological symptoms, maternal chronic pain, and parenting stress on children somatic complaints [60]. Dura and Beck [61] found that the children of mothers with chronic pain have more depression than two other control groups. Empirical evidence shows that parents with difficulty to regulate emotions and to express feelings, can inhibit the child’s ability to self-regulate his/her emotional states, thus influencing the bodily experience with the tendency to somatization [62]. Livingston, studying children of people with somatization disorders, assume that “all their children are at some risk for developing somatization disorder” and that “those who have begun having medically unexplained physical symptoms are likely to be experiencing the early stages of developing disorder”[63, 64].

Furthermore clinical and epidemiological evidence suggests that migraine co-occurs with psychopathology [65]

Merikangas [66] in her seminal paper on migraine and psychopathology finding a strong correlation between migraine, anxiety and depression, concluded: “Initial expression of anxiety, often in early childhood, was followed by the occurrence of migraine headaches and then by discrete episodes of depressive disorders in adolescence or in adulthood”. It is well known that headache in childhood is associated with several psychopathological states [56], in particular depression and anxiety [8, 9], epilepsy [67], sleep disorders [3], attention deficit hyperactivity disorder [6] which all seem to be related to headache.

The Young-Hunt Study [68], a large-scale population-based study in Norway assessing adolescent recurrent headache in relation to symptoms of anxiety and depression and behavioral problems, found that adolescents aged 12–17 years with headache have higher levels of anxiety and depression and that, especially between aged 15–17, higher levels of attention and conduct difficulties. Furthermore Arruda et al. [69] showed not only that children with headache are more likely to present with emotional symptoms, conduct problems, hyperactivity and peer problems, but also that they are at an increased risk of impaired psychosocial adjustment. The authors believe that the association between psychosocial adjustment problems and the burden caused by migraine may perpetuate both conditions.

Therefore the current theory explains headache like a complex multifactorial disorder with both predisposing genetic factors and environmental factors contributing to the attacks [70].

The impact of environmental factors on headache is furthermore well documented by many studies on the association between childhood adversity and chronic daily headache [71–75]. Some life events such as divorce, physical and sexual abuse are recognised as a risk factors for headache and its chronicity [76]. In particular abuse could have some effect at a neurobiological and epigenetic level, which could play a role in the pathogenesis of migraine [75].

The wide influence of environmental factors on migraine has led to consider migraine as a bio-psychosocial condition, caused by cognitive, emotional and environmental factors, as well as biological [77].

## Conclusions

In his seminal paper on psychosomatic, Alexander [78] defined a psychosomatic problem as “an individual psychodynamic adjustment that expresses itself in peculiar circumstances”. Psychosomatics represent an interaction between psychic and physical aspects with the former having an establishing effect on the latter. Mainly in younger children, an uncomfortable situation, a psychological problem or an emotional distress is rarely expressed directly but usually through physical symptoms. The body can be seen as mediator of the environmental stressors and psychological factors and the tendency to develop head pain when faced with strong sensory stimuli or physical or emotional stress might be the result of an evolved defence mechanism [79]. Indeed humans seem to be the only species that experiences migraine, so citing Loder [79] “vulnerability to recurrent headache may be the price we pay for consciousness”.

## Abbreviations

DMN: Default Mode Network
MwA: Migraine without Aura
MRI: Magnetic resonance imaging
WMLs: White Matter Lesions
CNS: Central Nervous System
CGRP: Calcitonin-Gene-Related Peptide
